# Hampstead Provident Dispensary

**Published:** 1890-03-22

**Authors:** 


### Passing Topics.

### HAMPSTEAD PROVIDENT DISPENSARY.

The committee of this useful and progressive institution, which appears to conduct its affairs with commendable discretion, express their regret in the report recently presented, "that the Hospital Saturday Fund Board have withheld their grant for 1889 on the ground that the dispensary does not give gratuitous aid or free letters." This appears not only an unfair but a somewhat ludicrous decision, and it may be pertinently asked whether the Saturday Fund delegates have stopped to consider what a provident dispensary is? They cannot object that the institution in question does not need help, because the accounts show that it does. The provident payments meet about one-half the expenses and no more, the balance being made up of subscriptions and donations, a grant from a local charity, and one from the Hospital Sunday Fund, the latter amounting to £52 1s. 8d., or about eight per cent. of the total receipts—no unsubstantial assistance, and indicating, as the committee have a right to assume, that the society's management is capable of passing satisfactorily through the ordeal of examination.

But if the dispensary is not self-supporting, on the other hand, those who make use of it do something according to their means to help themselves. And the fact that they voluntarily do that something furnishes a good solid reason for affording them the quantum of help needed to make up the deficit. To be logical in their refusal, the Saturday Fund Delegates ought to withhold help from every convalescent home which requires a weekly contribution from its inmates, and from every hospital which, according to a common custom, expects its patients to supply themselves with certain articles of diet not considered strictly necessaries.

Verily, managers of philanthropic institutions have a poor time. If they dispense their benefits for nothing, they are accused of pauperizing the working classes; when they encourage the poor to do something for themselves, a representative body of almoners thinks it right to refuse assistance because the work is not entirely gratuitous. It might be asked what is the Saturday Fund itself—ostensibly if not actually—but a provident fund to which the working classes are invited to contribute in order to secure hospital benefits in return? Its very avidity to secure "letters" shows that its grants are not gratuities.

It has always seemed to us that one great blot upon our system of hospital relief is that the patients are compelled to accept for nothing that which many of the more self-respecting among them would willingly pay for in part. A man earning thirty shillings or more a week, who brings his wife or child for out-treatment at the hospital, and not infrequently has been sent there by his doctor, is forbidden, presumedly in the interests of the profession, to pay the half-crown or five shillings he could well afford, and which would cover the expense incurred by the hospital and save his self-respect. Similarly, an in-patient is lodged, boarded, and treated for nothing, although, as sometimes happens, his income is actually unspent meanwhile.

Such cases are in a degree, a very insufficient degree of course, met by the provident dispensaries, and these institutions when well administered do as good work in their humble way as the great hospitals in theirs. We think the authorities of the Hampstead Dispensary would have been justified had they added a word of protest to their expression of regret at the withdrawal of the grant upon the ground that they are true to the principles and constitution of their society. It is a pity that the Saturday Fund organisation, to which everyone must wish well, should so often find itself in antagonism with educated opinion. There is a perversity and inconsistency about its methods which are inexplicable. How is it possible, for instance, to reconcile its attitude towards the Hampstead Dispensary with its treatment of St. John's Skin Hospital, the institution with the peculiar system of accounts where the item of "donations" is composed almost wholly of payments by patients?

Agricola.

